# Ophthalmological manifestations, visual outcomes, and treatment of electrical and lightning trauma: A Systematic Review

**DOI:** 10.1007/s00417-025-06844-3

**Published:** 2025-06-27

**Authors:** María A. Piedrahita, Andrés Felipe Pineda-Vanegas, Felipe Moreno-Mendoza, María Andrea Estévez-Flórez, Nicolas España-Isaza, Viviana Infante-Ortegón, Diana V. Rey-Rodríguez, Carlos Cifuentes-González, Laura Daniela Rodriguez-Camelo, Germán Mejía-Salgado, Alejandra de-la-Torre

**Affiliations:** 1 Department of Ophthalmology, Faculty of Medicine, Simón Bolívar Hospital, Universidad El Bosque, Bogotá, Colombia; 2 Faculty of Medicine, Universidad El Bosque, Optometry Program, UnBosque Visual and Ocular Health Research Group, Bogotá, D.C Colombia; 3 Neuroscience (NEUROS) Research Group, Institute of Translational Medicine (IMT), School of Medicine and Health Sciences, Neurovitae Research Center, Universidad del Rosario, Bogota, Colombia; 4 Ophthalmology Interest Group-Universidad del Rosario. (OIG UR). Neuroscience (NEUROS) Research Group, Institute of Translational Medicine (IMT), School of Medicine and Health Sciences, Neurovitae Research Center, Universidad del Rosario, Bogota, Colombia; 5 Centre of Excellence in Eye Inflammation, Colombian Visual Science and Translational Eye Research Institute (CERI), Bogotá, Colombia

**Keywords:** Electrical trauma, High-voltage injury, Trauma, Ophthalmological complications, Visual outcomes

## Abstract

**Purpose:**

Electrical and lightning injuries are rare but carry significant risks for ocular complications. This systematic review aims to synthesize data on ocular manifestations, visual outcomes, and treatment approaches following such traumas.

**Methods:**

A comprehensive literature search was conducted up to August 21, 2023, utilizing PubMed, Embase, and Web of Science databases. Observational studies, including case reports, case series, and cross-sectional studies involving patients with electrical and lightning injuries, were included. Quality and risk of bias were assessed using the Hoy et al. tools for cross-sectional studies and the Hassan Murad et al. recommendations for case reports and series. Data on ocular manifestations, visual outcomes, and administered treatments were extracted. Manifestations were compared across high-voltage (≥ 1000 V) and low-voltage (< 1000 V) exposures. Visual outcomes were classified into no visual impairment (Best Corrected visual acuity [BCVA] better than 0.4 LogMAR), any vision impairment (BCVA of 0.4 LogMAR or worse), and legal blindness (BCVA of 1.0 LogMAR or worse). This review is registered with PROSPERO (CRD42023453495).

**Results:**

The review included 71 studies comprising 183 patients. Cataracts were the most common anterior segment finding (30.05%), and macular cysts were the predominant posterior segment finding (10.38%). Visual outcomes reported for 56 patients showed that 51.35% experienced some degree of visual impairment, with 33.92% classified as legally blind. High-voltage exposure was associated with a higher frequency of cataracts (82.35% vs 37.50%) and macular cysts (11.76% vs. 0%) compared to low-voltage injuries. Patients with legal blindness more frequently exhibited cataracts (73.68% of legally blind patients vs. 61.29% with some visual impairment vs. 56.0% with no visual impairment) and macular cysts (26.32% of legally blind patients vs. 12.0% with no visual impairment). Surgical interventions such as phacoemulsification (15.85%) and vitrectomy (6.02%) were conducted.

**Conclusion:**

Electrical and lightning injuries can lead to significant ocular complications; the frequency of those complications varies according to the voltage of exposure and the resulting visual outcomes, occurring more frequently in patients exposed to high voltages and those who are legally blind.

**Supplementary Information:**

The online version contains supplementary material available at 10.1007/s00417-025-06844-3.

## Key Messages

*****What is known***:**

- Electrical and lightning injuries are rare but can lead to significant ocular complications. Most existing literature on ocular trauma resulting from electrical and lightning injuries is derived from case reports, which limits the ability to calculate the frequency of specific ophthalmological manifestations.

*****What is new***:**

- This review identifies cataracts and macular cysts as the most frequent ocular complications associated with high-voltage trauma, emphasizing their predominance in the anterior and posterior segments, respectively.
- Visual outcomes vary significantly, with over half of the patients experiencing some level of visual impairment and nearly one-third classified as legally blind, particularly those exposed to high-voltage incidents.
- Phacoemulsification and vitrectomy can be required following these traumas.

**Supplementary Information:**

The online version contains supplementary material available at 10.1007/s00417-025-06844-3.

## Introduction

Electrical and lightning trauma are uncommon, comprising around 4% of all trauma incidents [1]. Tissue damage resulting from an electric shock can occur due to the passage of currents through tissues, electrothermal or arc burns from currents that pass externally to the body, and/or thermal burns from ignited clothing or nearby objects [1]. The severity of these injuries depends on various factors, including the voltage level, the type of current (alternating or direct), the path the current takes through the body, and the duration of contact [2]. Electrical burns are typically classified as high-voltage (≥ 10,000 V) or low-voltage (< 1000 V) [1, 2]. These injuries can lead to various manifestations across different systems, including skin, skeletal, muscular, respiratory, cardiac, neurological, and ocular damage. Notably, about 15% of affected individuals experience involvement in multiple systems [2, 3].

Ophthalmological manifestations resulting from electrical trauma exhibit a wide range of clinical features, which can appear immediately or develop several years post-incident [4]. The most reported ocular complications include cataracts and damage to the ocular surface, such as eyelid edema, conjunctival chemosis, and epithelial keratitis [4]. However, electrical trauma can also result in vision-threatening conditions like optic neuropathy and retinal complications, including macular edema and holes [4].

Compared to other types of ocular burns, such as chemical and thermal burns [5, 6], the characterization of ophthalmological manifestations in electrical trauma is limited, as the initial burn injury is often life-threatening, and ophthalmological assessment is not performed [4]. Consequently, most available information is primarily from case reports, series, and narrative reviews. This systematic review summarizes information on ocular manifestations, visual outcomes, and therapeutic interventions following electrical and lightning injuries. The findings will be helpful to eye care professionals in assessing patients who have experienced this type of trauma.

## Methods

This systematic review followed the ‘Preferred Reporting Items for Systematic Review and Meta-analysis (the'PRISMA'statement) [7] **(Supplementary Material 1).** This review was registered in PROSPERO under the reference CRD42023453495. Institutional review board approval was not required, as this study is based on data available in the public domain.

### Search strategy

**Fig. 1:**
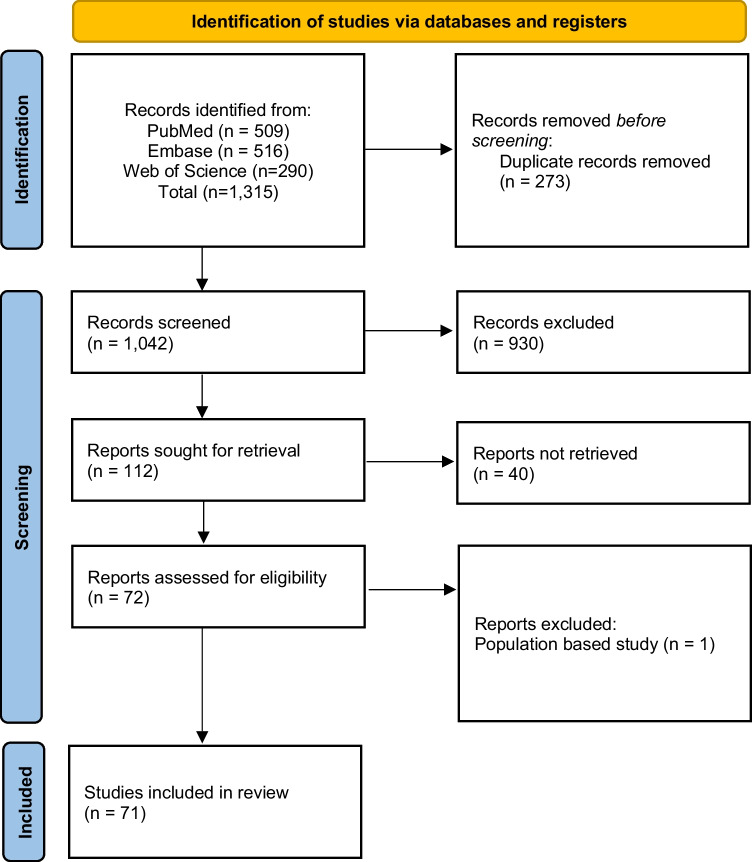
Prisma Flowchart

A systematic literature search was conducted using PubMed, Embase, and Web of Science on August 21, 2023. The search algorithm included a combination of terms reflecting the events of interest (electrical traumas) and (ocular manifestations, treatments, and visual outcomes) using the following search strategy:'eye'[MeSH Terms] OR'eye'[All Fields] OR retina [Title/Abstract] OR"cornea"[Title/Abstract] OR"cataract"[Title/Abstract] OR"optic nerve"[Title/Abstract] OR"macula"[Title/Abstract] OR"uvea"[Title/Abstract] OR"anterior segment"[Title/Abstract] OR"posterior segment"[Title/Abstract]) AND “electric injuries” [MeSH Terms] OR “electric” [All Fields] OR'electric injuries'[All Fields] OR “electric” [All Fields] OR “electric injury” [All Fields] OR “burns, electric” [MeSH Terms] OR “burns” [All Fields] OR “electric burns” [All Fields] OR “burn”[All Fields]) OR “electric burn” [All Fields]. The search strategies were modified to meet the criteria and terms of each database. On August 23, 2024, the search strategy was conducted again to identify recent articles published. The search process was documented following the PRISMA statement (Fig. 1).

### Study eligibility criteria

Primary observational studies, including case reports, case series, case-control studies, cross-sectional studies, and cohort studies were included. There were no restrictions on language or publication year. Google Translate was utilized to select and extract information from articles written in languages other than English and Spanish [8]. Excluded from the review were non-full-text articles, studies conducted in non-human species, and secondary data sources such as narrative, systematic, or scoping reviews.

### Patient inclusion and exclusion criteria

Patients of all ages and genders with accidental or provoked electrical trauma of any voltage or lightning trauma, with ophthalmological assessment were included. Conversely, patients with burns resulting from other sources, such as thermal, solar, or welding trauma, were excluded.

### Study selection

Search results from each database were downloaded in RIS format and managed using Zotero®. Duplicates were then identified and removed using the duplicate detection function in both Zotero® and the Rayyan® research collaboration platform. After eliminating duplicates, articles were randomly assigned to and independently screened by three pairs of reviewers (AFPV, AFMM, MAEF, NEI, VIO, DRR) for a title and abstract review, followed by a full-text review. After this review, articles were categorized as “included”, “excluded”, or “in doubt” and documented in a Microsoft Excel® database. Any discrepancies in eligibility were resolved by consensus among the authors, and if unresolved, an ophthalmology specialist was consulted. The selection process is illustrated in Fig. 1.

### Data extraction

The included articles were downloaded and recorded in a Microsoft Excel® spreadsheet, each assigned a unique identification number. Six reviewers (AFPV, AFMM, MAEF, NEI, VIO, DRR) carried out data registration, which was subsequently validated by two independent reviewers (GMS, LDRC). The data extraction database included the following information: article title, publication year, DOI, author details, patient age, sex, ophthalmological manifestations, time to ophthalmological assessment, voltage, visual outcomes, and treatment approaches.

### Risk of bias assessment

The risk of bias assessment was conducted independently by three pairs of reviewers (AFPV, AFMM, MAEF, NEI, VIO, DRR) and subsequently validated by two independent reviewers (GM-S, LDRC) using validated tools appropriate to the methodological design of the articles. For cross-sectional studies, the Hoy et al. modified tool was employed [9]. This tool comprises 10 items addressing four domains and a summary risk of bias assessment. The items included: 1) target population, 2) sampling frame representation, 3) sample selection, 4) likelihood of nonresponse, 5) data collection source, 6) case definition, 7) parameters measurement, 8) data collection consistency, 9) follow-up period, and 10) appropriateness of numerator and denominator for the parameter of interest. The four domains assessed were selection, nonresponse, measurement, and analysis bias. Each “yes” response counted for one point. External validity was rated as “high” for scores of 0–1, “some concerns” for scores of 2, and “low” for scores of 3. Similarly, internal validity was rated as “high” for scores of 0–2, “some concern” for a score of 3, and “low” for a score of 4. Studies were considered to have a “high risk” of bias if either domain (internal or external validity) was rated as having a “high risk of bias.”[9].

The Hassan Murad recommendations were followed for case reports and case-series studies, consisting of four general items: 1) population selection, 2) ascertainment of exposure or outcome, 3) causality, and 4) sufficient reporting details [10]. The scoring was simplified: a “yes” answer was assigned a “low risk of bias”, a “no” answer was assigned a “high risk of bias, and a “not applicable”' answer was assigned “some concerns.” A study was considered to have a “low risk of bias” if all items were scored as low risk, “some concerns” if 1–2 items had a high risk of bias, and “high risk” if three or more items were scored as high risk of bias.

### Data synthesis and analysis

A narrative description was performed in **Supplementary Material 2** for the qualitative analysis, describing the main ophthalmological findings, baseline visual acuity, visual outcomes, and medical and surgical treatments in the identified cases. Subsequently, the ophthalmologic findings were analyzed using a Microsoft Excel database to determine each manifestation frequency. Calculations were performed at the patient level, meaning that if a manifestation was present in at least one eye, it was marked as present; the results are presented as absolute frequencies and percentages. Further subgroup analyses were conducted on articles reporting information based on voltage severity, classified as high-voltage (≥ 1000 V) or low-voltage (< 1000 V), and final best-corrected visual acuity (BCVA) based on the better-seeing eye. Visual outcomes were classified as no visual impairment (final BCVA better than 20/50 Snellen chart [< 0.4 logMAR]), any vision impairment (final BCVA of 20/50 or worse Snellen chart [≥ 0.4 logMAR]), or legal blindness (final BCVA of 20/200 or worse [≥ 1.0 logMAR]).

## Results

### Study selection

Initially, 1,315 records were identified as potentially relevant studies. Among these, 273 were duplicates and subsequently eliminated. During the initial screening, 930 articles were excluded based on title and abstract, leaving 112 reports sought for retrieval. From those, 72 were assessed for eligibility. However, one population-based study of patients with electrical trauma was excluded because the ophthalmological assessment was not described comprehensively [11]. Therefore, 71 studies involving 183 patients were included. (Fig. 1).

### Study and patient characteristics

The included studies comprised 66 case reports, four case series, and one cross-sectional study. Among the case reports, seven were rated as having a “low risk of bias,” 15 were categorized as having a “high risk of bias,” and 44 were deemed to have “some concerns.” Of the four -case series, two were classified as having a “high risk of bias,” and two was rated as having “some concerns.” The cross-sectional study was determined to have “some concerns” **(Supplementary Material 3).** Geographically, 33 studies were reported in Asia, 22 in North America, 11 in Europe, and five in South America. Overall, 45/71 studies (63.38%) documented anterior segment findings, 51/71 (71.83%) reported posterior segment manifestations, 49/71 (69.01%) included information on the treatment administered, and 42/71 (59.15%) reported visual outcomes(Table 1).

Regarding patient demographics, 91.80% were male (168/183), and 6.55% were female (12/183), with three patients not reporting their sex. Lightning strikes accounted for 14.75% of the cases (27/183). Electrical trauma occurred during outdoor activities in 18.03% of patients (33/183), while 11.48% (21/183) sustained injuries during work-related activities. The site of current contact was cranial in 13.11% of the cases (24/183) and non-cranial for 7.64% of the cases (14/183); the latter includes the right hand, right thumb, left arm, among others. 144 cases did not report the site of current contact. The median time from injury to ophthalmological evaluation was 28 days, with an interquartile range (IQR) of 115.5 days. (Table 1).

**Table 1:** Study characteristics

Author, year	Country	Sex	Type of study	Mechanism	Voltage (V)	Time to service (days)	Anterior segment findings	Posterior segment findings	Treatment documented	Visual outcomes documented
Cherrington et al. 1999 [12]	USA	M	Case Report	Struck by lightning while skiing	ND	0	Yes	No	Yes	Yes
Cazabon et al. 2000 [13]	UK	F	Case Report	Struck by a bolt of lightning while walking on a seaside cliff	ND	4	No	No	Yes	Yes
Sommer et al. 2004 [14]	Denmark	F	Case Report	Stroke of lightning while walking bare isthmus of land	ND	7	Yes	No	Yes	Yes
Lin et al. 2002 [15]	Taiwan	M	Case Report	Struck on the left forehead by a bolt of lightning	ND	2	Yes	Yes	Yes	Yes
Korn et al. 2014 [16]	USA	M	Case Report	Camps site was struck by lightning, man was asleep in a tent burn	14,000	120	Yes	Yes	Yes	yes
Espaillat et al. 1998 [17]	Dominican Republic	M	Case Report	Lightning	ND	7	Yes	Yes	Yes	Yes
Manrique-Cerrillo et al. 2004 [18]	México	M	Case Report	Electrical burn	ND	60	No	Yes	No	Yes
Rivas-Aguiño et al. 2006 [19]	Venezuela	F	Case Report	Sleeping with the left shoulder and face in contact with the floor of a rural house that got struck by lightning	ND	60	Yes	Yes	No	Yes
Whelan et al. 1988 [20]	Germany	M	Case Report	Contact with a direct current cable	15,000	10	Yes	No	Yes	No
Kubilius et al. 2012 (27)	Lithuania	ND	Case Series	Struck by lightning under some trees	ND	ND	No	Yes	No	Yes
Campo et al. 1984 [21]	USA	F	Case Report	Struck by lightning while horseback riding	ND	14	Yes	Yes	No	Yes
Hunt et al. 2000 [22]	USA	3M	Case Series	1: Struck on the left temple by a bolt of lightning while mountain climbing 2: Man was asleep in a tent when lightning struck 3: Lightning struck a pier, where the patient was	ND	ND	Yes	Yes	Yes	Yes
Givner et al. 1956 [23]	USA	M	Case Report	Fell on the third rail of the subway and hit the region of his right eye. The third rail carries 650 volts of D.C. current	650	28	Yes	No	Yes	Yes
Wainwright et al. 1994 [24]	USA	M	Case Report	Contact with a power line.	7,200	14	Yes	Yes	No	Yes
Sizman et al. 2020 [25]	Turkey	M	Case Report	Lightning strike	ND	180	Yes	Yes	Yes	Yes
Mishulin et al. 2020 [26]	USA	M	Case Report	Electrocuted with 12,000 V	12,000	28	No	Yes	Yes	No
Almari et al. 2020 [27]	Saudi Arabia	M	Case Report	Contact with a current of 1200V	1,200	2	Yes	Yes	Yes	Yes
Korkmaz et al. 2018 [28]	Turkey	M	Case Report	Accidental exposure to high-voltage electrical current	ND	60	No	Yes	Yes	Yes
Pradhan et al. 2020 [29]	Nepal	4 F 3M	Case Series	1: Side lightning strike 2: Ground current 3: Direct lightning strike 4: Direct lightning strike 5: Direct lightning strike 6: Direct lightning strike 7: Direct lightning strike	ND	12	Yes	Yes	Yes	Yes
Yadav et al. 2020 [30]	India	M	Case Report	Electric transmission cable accidentally fell on his head	11,000	180	No	Yes	Yes	Yes
Harris et al. 2019 [31]	USA	M	Case Report	Lightning strike injury to both eyes	ND	14600	No	Yes	Yes	Yes
Kumawat et al. 2017 [32]	India	M	Case Report	Contact with a high-tension wire (11000 V) on his hands	11,000	540	Yes	Yes	Yes	Yes
Liu et al. 2016 [33]	USA	F	Case Report	Struck by lightning directly to the right forehead while hiking	ND	14	No	Yes	No	Yes
Izzy et al. 2014 [34]	USA	M	Case Report	Electrocuted by an electric power supply	380	ND	No	Yes	Yes	No
Baranwal et al. 2012 [35]	India	M	Case Report	Electric injury due to direct contact of high-tension wire	ND	365	Yes	No	Yes	Yes
Bayar et al. 2013 [36]	Turkey	M	Case Report	Electrocuted while fixing a high-voltage transmission line tower	ND	14	No	Yes	Yes	Yes
Toprak et al. 2014 (43)	Turkey	F	Case Report	Indirect lightning accident 25 years earlier while she was ironing at home	ND	9125	No	Yes	No	No
Tandon et al. 2014 [37]	India	M	Case Report	Electrocution with overhead transmission wires	20,000	14	Yes	Yes	Yes	Yes
Armstrong et al. 2010 [38]	USA	F	Case Report	Lightning	ND	0.04	No	Yes	No	No
Rajagopal et al. 2010 [39]	Canada	M	Case Report	Following accidental contact with a high-tension electrical wire	440	4	No	Yes	Yes	No
Rao et al. 2009 [40]	India	F	Case Report	Struck by lightning	ND	1	Yes	Yes	No	No
Khan et al. 2009 [41]	Arab emirates	M	Case Report	Electrocution (high voltage cable)	4,000	10	No	Yes	No	No
Grewal et al. 2007 [42]	India	M	Case Report	Contact with an electric transmission wire	11,000	30	Yes	No	Yes	Yes
Liyanage et al. 2006 [43]	UK	M	Case Report	Accidental electrical shock to his left forearm	230	273	Yes	Yes	No	Yes
Mutlu et al. 2004 [44]	Turkey	F	Case Report	High-voltage electrical injury	10000	3	Yes	No	Yes	Yes
Dinakaran et al. 1998 [45]	UK	M	Case Report	Lightning strike when he was using a telephone at his home during a thunderstorm	ND	1095	No	No	Yes	No
Ranjan et al. 2017 [46]	India	M	Case Report	Domestic accident with hand being the entry point and feet being the exit point	ND	90	No	Yes	No	Yes
Dolphin et al. 1992 [47]	USA	M	Case Report	A railroad worker, inadvertently engaged a third rail, which was carrying 700 V of electricity	700	90	Yes	No	No	Yes
Khazaei et all, 2022 [48]	Iran	M	Case Report	Electrical injury in his workplace	400	1461	No	Yes	No	Yes
Dhillon et al, 2015 [49]	UK	F	Case Report	Indirectly struck by lightning	ND	4	No	Yes	No	Yes
Moon et al. 2005 [50]	USA	F	Case Report	Struck by lightning while she was in a bamboo house during a missionary training	ND	1461	No	No	Yes	Yes
Choe et al. 2019 [51]	South Korea	M	Case Report	High-voltage electric shock injury	22,000	60	No	Yes	Yes	Yes
Faustino et al. 2014 [52]	Brazil	M	Case Report	Electrical shock at his workplace caused by a high-voltage wire	ND	14	Yes	Yes	Yes	Yes
Hashemi et al. 2008 [53]	Iran	M	Case Report	Shock from a high-voltage alternating current power line cable	1,0000	30	No	Yes	Yes	Yes
Sony et al. 2005 [54]	India	M	Case Report	Accidentally touched an exposed electrical wire.	ND	ND	No	Yes	Yes	Yes
Ouyang et al. 2014 [55]	China	M	Case Report	High-voltage electrical injury at work	35,000	ND	Yes	Yes	Yes	Yes
Lakosha et al. 2009 [56]	Canada	F	Case Report	Swimming near sailboat when the sailboat’s mast struck a high-voltage power electric line	ND	120	Yes	No	Yes	No
Gupta et al. 2009 [57]	India	M	Case Report	Struck by lightning while he was walking on a street.	ND	1461	Yes	No	Yes	No
Al Rabiah et al. 1987 [58]	UK	M	Case Report	Contact with a high-tension electrical cable	33,000	4	Yes	Yes	No	Yes
Biro et al. 1994 [59]	Hungary	M	Case Report	Contact with high-tension electric pole	22,000	304	Yes	Yes	Yes	Yes
Sharma et al. 2019 [60]	India	M	Case Report	Electric shock: the point of contact was the right thumb	440	60	Yes	Yes	Yes	Yes
Reddy et al. 1999 [61]	India	M	Case Report	Direct contact with a high-voltage	11,000	14	Yes	Yes	Yes	Yes
Duman et al. 2015 [62]	Turkey	F	Case Report	Unspecified electrocution	ND	30	Yes	Yes	Yes	No
Peñaranda C, 2016 [63]	China	M	Case Report	Electric burn of 11,000 volts due to inadvertent contact	10,000	120	Yes	Yes	Yes	Yes
Zhang et al. 2023 [64]	USA	M	Case Report	Unspecified electrocution	280	0.08	Yes	Yes	Yes	No
Bae et al. 2013 [4]	South Korea	101 M 1 F	Cross-sectional	ND	200-2,0 000	ND	Yes	Yes	No	No
Pineda-Vanegas et al. 2024 [65]	Colombia	M	Case report	Third-degree electrical burns	ND	ND	Yes	No	Yes	Yes
Venkateswaran, et al 2018 [66]	USA	F	Case report	Struck by lightning as a child	ND	ND	Yes	No	No	No
Rishi et al. 2016 [67]	India	M	Case report	Struck by lightning	ND	304	No	Yes	No	No
Walkow et al. 2011 [68]	Germany	M	Case report	Train accident while providing first aid to injured passengers	ND	ND	Yes	Yes	Yes	Yes
Boozalis et al. 1991 [69]	USA	1F 2M 2 ND	Literature Review and Report of Cases	1: Electric company injury 2: Struck by a fallen power linc 3: Struck in the forchead with a wire 4: Struck ini the face by a "high-voltage power line" 5: Struck by a power line.	1: 14400 2: ND 3: 7200 4: ND 5: ND	ND	Yes	Yes	Yes	Yes
Zhang et al. 2024 [70]	USA	M	Case report	Electrical burn with a direct current	1500	0.08	Yes	Yes	Yes	Yes
Yi et al. 2024 [71]	China	M	Case report	Struck by lightning in a tent	ND	0.06	Yes	Yes	Yes	Yes
Valera-cornejo et al. 2020 [72]	Mexico	M	Case report	Head accidentally touched an exposed electrical wire	ND	120	Yes	Yes	Yes	Yes
Khadka et al. 2021 [73]	Nepal	2 M	Case series	1: Struck by lightning 2: Struck by lightning	ND	1: 0.09 2: 12	Yes	Yes	Yes	Yes
Datta et al. 2002 [74]	India	M	Case report	Injury by lightning	ND	12	Yes	No	Yes	Yes
Bienfang et al. 1980 [75]	USA	M	Case report	Accidental touch of a "third rail" of a subway	ND	0	Yes	Yes	Yes	Yes
Hashemi et al. 2008 [53]	Iran	M	Case report	Electrical shock	10000	36	Yes	No	Yes	Yes

*F*, Female; *M*, Male; *ND*, No data; *USA*, United States of America

### Ophthalmological manifestations, visual outcomes, and treatment

Among the 183 patients, 11.48% (21/183) presented with eyelid erythema and swelling. Regarding anterior segment findings, the most common manifestation was cataract, observed in 55 patients (30.05%). Among patients with cataracts, 32.72% (18/55) had posterior subcapsular cataracts, 25.45% (14/55) had anterior subcapsular cataracts, 10.91% (6/55) had cortical cataracts, and 7.27% (4/55) had nuclear cataracts. Other prevalent anterior segment findings included superficial punctate keratitis in 12.02% (22/183), anterior uveitis in 9.28% (17/183), corneal edema in 6.56% (12/183), conjunctivitis in 5.46% (10/183), chemosis in 3.82% (7/183), and keratitis in 3.82% (7/183). (Table 2).

As for posterior segment findings, the most common were macular cysts, affecting 10.38% of patients (19/183), followed by macular hole in 8.74% (16/183), retinal pigment epithelium (RPE) disruption in 7.65% (14/183), retinal detachment, macular edema, and retinal atrophy each in 4.38% (8/183), posterior vitreous detachment and retinal hemorrhage each in 3.28% (6/183), optic nerve atrophy, optic neuritis and retinal thickening each in 2.73% (5/183), and retinoschisis in 0.55**%** (1/183). (Table 2).

**Table 2:** Ophthalmological manifestations, visual outcomes, and treatment of the 183 patients with electrical trauma reported in the literature until August 2024

	Ophthalmological manifestations	Number of patients	%
Eyelids Findings	Eyelid erythema, swelling	21	11.48
Anterior Segment Findings	Any type of cataract	55	30.05
	Superficial punctate keratitis	22	12.02
	Posterior subcapsular cataract	18	9.84
	Anterior subcapsular cataract	14	7.65
	Anterior uveitis	17	9.28
	Corneal edema	12	6.56
	Conjunctivitis	10	5.46
	Chemosis	7	3.83
	Cortical cataract	6	3.28
	Nuclear cataract	4	2.19
	Keratitis	7	3.83
	Ocular hypertension	4	2.19
	Symblepharon	3	1.64
	Intermediate uveitis	1	0.55
	Corneal scarring	2	1.09
	Lens dislocation	1	0.55
	Necrosis of the iris, ciliary epithelium	2	1.09
	Coagulative necrosis of the cornea, sclera and anterior uveal tract	1	0.55
	Corneal descompensation	1	0.55
Posterior segment findings	Macular cyst	19	10.38
	Macular hole	16	8.74
	RPE disruption	14	7.65
	Retinal detachment	8	4.38
	Macular edema	8	4.38
	Retinal atrophy	8	4.38
	Posterior vitreous detachment	6	3.28
	Retinal thickening	5	2.73
	Retinal hemorrhage	6	3.28
	Optic nerve atrophy	5	2.73
	Unspecified macular lesion	2	1.09
	Optic neuritis	2	1.09
	Posterior uveitis	1	0.55
	Retinoschisis	1	0.55
	Central retinal vein occlusion	1	0.55
	Purtscher-like Retinopathy	1	0.55
	Electric shock maculopathy	1	0.55
	Choroidal atrophy	1	0.55
	Retinal and choroidal necrosis	1	0.55
Visual outcomes			
No vision impairment (Final BCVA <20/50 Snellen chart, <0.4 LogMAR)		25	13.66
Any vision impairment (Final BCVA >20/50 Snellen chart, >0.4 LogMAR)		31	16.93
Legal blindness (Final BCVA >20/200 Snellen chart, >1.0 LogMAR)		19	10.38
No data		127	69.39
Treatment strategies			
Any topical drops		26	14.20
Cycloplegic		15	8.20
Corticosteroids		21	11.48
Hypotensive		2	1.09
Surgical intervention		30	16.39
Phacoemulsification		29	15.85
Vitrectomy		11	6.02
Iridotomy		1	0.55
Trabeculectomy		1	0.55
Evisceration		2	1.09
Scleral buckle		1	0.55
Membrane peeling		1	0.55

RPE: Retinal pigment epithelium, BCVA: Best corrected visual acuity

Final visual acuity was reported for 56 patients with a mean LogMAR of 0.85 (Standard deviation (SD) = 1.01). Of them, 25/56 (44.65%) had no visual impairment, while 31/49 (55.35%) experienced some degree of visual impairment, including 19/56 (33.92%) who were legally blind. Notably, 69.39% (127/183) of patients did not report the final visual acuity.

Twenty-six patients received topical drops, including corticosteroids (11.48%, 21/183), cycloplegics (8.20%, 15/183), and hypotensive drops (1.09%, 2/183). Additionally, seven patients (3.83%) received systemic corticosteroids, and six (3.28%) were treated with antibiotics. Surgical interventions were performed on 30 patients, with phacoemulsification being the most common (15.85%, 29/183), followed by vitrectomy (6.02%, 11/183) and evisceration (1.09%, 2/183). Other procedures included iridotomy, trabeculectomy, scleral buckle, and membrane peeling, each performed in 0.55% of patients (1/183). (Table 2).

### Distribution of ophthalmological manifestations regarding final visual acuity

Cataracts were more common in patients with any grade of vision impairment at the final follow-up (61.29% [19/31]) compared to those with no vision impairment (56.0% [14/25]). Findings like macular cysts, macular holes, and RPE disruption were more prevalent in patients with any grade of visual impairment compared to those without vision impairment (38.71%% vs. 12.00% for macular cysts, and 29.03% vs. 12.00% for macular holes and RPE disruption). (Table 3).

**Table 3:** Distribution of Ophthalmological Manifestations Regarding Final Visual Acuity

	Final visual acuity without vision impairment a n= 25		Final visual acuity with any grade of vision impairment b n=31		Final visual acuity with legal blindness c n=19	
Cataract	14	56.00	19	61.29	14	73.68
Superficial punctate keratitis	2	8.00	3	9.68	2	10.53
Conjunctivitis	2	8.00	1	3.23	2	10.53
Chemosis	2	8.00	3	9.68	2	10.53
Corneal edema	4	16.00	4	12.90	3	15.79
Posterior vitreous detachment	0	0.00	2	6.45	2	10.53
Ocular hypertension	1	4.00	3	9.68	2	10.53
Keratitis	4	16.00	1	3.23	0	0.00
Symblepharon	0	0.00	3	9.68	3	15.79
Corneal descompensation	0	0.00	1	3.23	1	5.26
Lens dislocation	1	4.00	0	0.00	0	0.00
Anterior uveitis	4	16.00	3	9.68	2	10.53
Intermediate uveitis	1	4.00	0	0.00	0	0.00
Posterior uveitis	1	4.00	0	0.00	0	0.00
Retinoschisis	0	0.00	0	0.00	0	0.00
Central retinal vein occlusion	1	4.00	0	0.00	0	0.00
Retinal thickening	2	8.00	1	3.23	1	5.26
Retinal atrophy	1	4.00	4	12.90	4	21.05
Retinal detachment	0	0.00	5	16.13	5	26.32
Retinal hemorrhage	2	8.00	2	6.45	1	5.26
Purtscher-like retinopathy	1	4.00	0	0.00	0	0.00
Optic neuritis	0	0.00	0	0.00	0	0.00
Optic nerve atrophy	0	0.00	2	6.45	2	10.53
Macular edema	1	4.00	3	9.68	0	0.00
Macular cyst	3	12.00	12	38.71	5	26.32
Macular hole	3	12.00	9	29.03	4	21.05
RPE disruption	3	12.00	9	29.03	7	36.84
Electric shock maculopathy	1	4.00	0	0.00	0	0.00
Coagulative necrosis of the cornea, sclera and anterior uveal tract	0	0.00	1	3.23	1	5.26
Central metamorphopsia	0	0.00	2	6.45	2	10.53
Choroidal atrophy	0	0.00	1	3.23	1	5.26

RPE: Retinal pigment epithelium, BCVA: Best corrected visual acuity

^a^: final BCVA better than 20/50 Snellen chart [<0.4 logMAR]

^b^: final BCVA of 20/50 or worse Snellen chart [≥0.4 logMAR]

^c^: final BCVA of 20/200 or worse [≥ 1.0 logMAR]

### Distribution of findings regarding electrical voltage

Among 23 patients with available data on both voltage exposure and ophthalmological manifestations, Some manifestations presented with a higher frequency in the group of high voltage (≥ 1,000 V) compared with patients with low electrical voltage (< 1,000 V), including cataracts (882.35%% vs. 37.50%), superficial punctate keratitis (11.76% vs. 0%), chemosis (5.88% vs. 0%), ocular hypertension (11.76% vs. 0%), lens dislocation (5.88% vs. 0%), retinal atrophy (5.88% vs. 0%), optic nerve atrophy (5.88% vs 0%), macular edema (5.88% vs 0%), and macular cyst (11.76% vs 0%). Conversely, some manifestations were higher in the low electrical voltage group, like corneal edema (25.00% vs. 0%), symblepharon (12.50% vs. 0%), retinal thickening (12.50% vs. 0%), optic neuritis (12.50% vs. 0%), among others (Table 4).

**Table 4:** Distribution of ophthalmological manifestations regarding electrical voltage

	Low voltage <1,000 V n=8		High voltage >1.000 V n=17	
Cataract	3	37.50	14	82.35
Superficial punctate keratitis	0	0.0	2	11.76
Keratitis	1	12.5	0	0.0
Chemosis	0	0.0	1	5.88
Corneal edema	2	25.0	0	0.0
Posterior vitreous detachment	1	12.5	0	0.0
Ocular hypertension	0	0.0	2	11.76
Symblepharon	1	12.5	0	0.0
Corneal descompensation	1	12.5	0	0.0
Lens dislocation	0	0.0	1	5.88
Anterior uveitis	2	25.0	3	17.65
Central retinal vein occlusion	1	12.5	0	0.0
Retinal thickening	1	12.5	0	0.0
Retinal atrophy	0	0.0	1	5.88
Retinal detachment	1	12.5	1	5.88
Retinal hemorrhage	3	37.5	1	5.88
Purtscher like retinopathy	1	12.5	0	0.0
Optic neuritis	1	12.5	0	0.0
Optic nerve atrophy	0	0.0	1	5.88
Macular edema	0	0.0	1	5.88
Macular cyst	0	0.0	2	11.76
Macular hole	1	12.5	3	17.65
RPE disruption	2	25.0	3	17.65

V: Volts, RPE: Retinal pigment epithelium

## Discussion

**Fig. 2:**
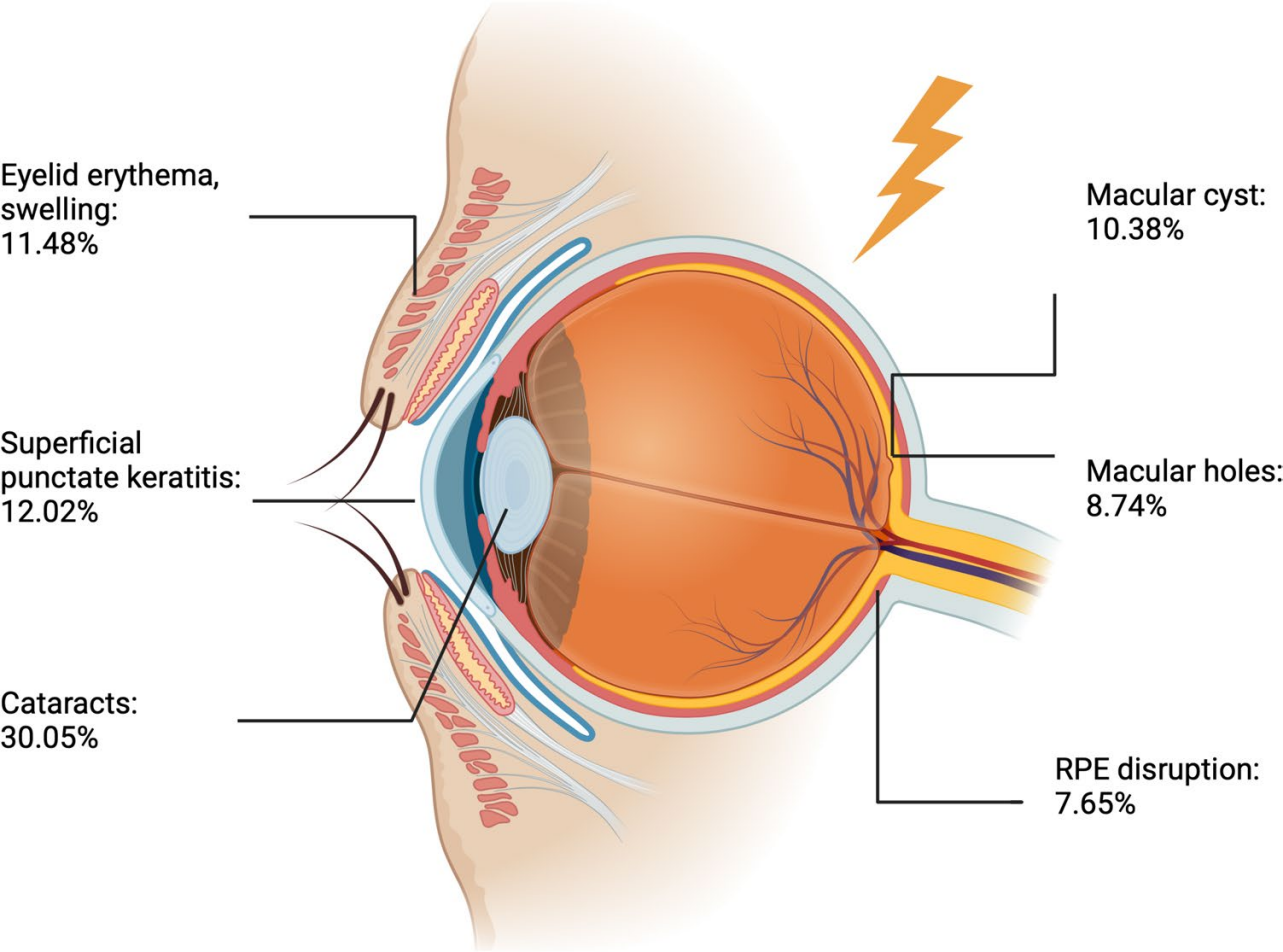
Principal ophthalmological findings in electrical and lightning trauma

This study summarizes the ocular manifestations (Fig. 2), visual outcomes, and therapeutic interventions following electrical and lightning injuries. The current literature on this topic is limited and primarily consists of case reports. Therefore, the findings presented in this review offer valuable insights for clinicians in identifying ocular manifestations in patients who present to the ophthalmology department after experiencing electrical trauma.

Consistent with previous studies, young men are the most affected demographic, with most incidents occurring in the workplace [4]. Similarly, this study found a predominance of male patients; however, electrical trauma was more related to outdoor activities than during work-related activities, underscoring the need for safety policies not only in workplaces [76] but also in other environments [77]. It is crucial to reinforce the importance of protective measures, such as inspecting tools and power cords before use, avoiding the combination of water and electricity, and following the manufacturer's instructions for power tools, appliances, and equipment to prevent electrical trauma [77].

The most commonly observed complication in this study was cataract formation, which aligns with previous literature identifying it as the most frequent complication [78]. However, Bae et al. reported that punctate corneal epithelial erosions were the most common finding [4]. This discrepancy may be attributed to the demographic differences between the populations studied. Bae et al. evaluated 102 patients who presented with ocular issues among 557 electrical burn patients treated at the Hallym Burn Center between 2004 and 2010, with all ophthalmic examinations conducted within 48 h of consultation. In contrast, the present study included cases worldwide, with a longer time of presentation (median 28 days IQR 115.5).

Regarding the posterior segment, macular holes and optic neuritis are commonly reported complications [78]. In this study, macular cysts were the most prevalent finding, followed by macular holes. The frequent involvement of the macula may be explained by its high concentration of melanin compared to other ocular tissues, which increases electrical resistance and reduces heat dissipation. This combination can lead to the development of edema, holes, and cysts, as observed in these cases [79].

The onset of cataracts following electrical trauma varies widely, ranging from immediate post-injury development to several years later. This prolonged latency raises concerns regarding the causative role of electrical injury in cataract formation [80]. For instance, Ibrahim Toprak and Volka Yaylali reported a case of bilateral visual loss occurring 25 years after an indirect lightning injury, underscoring the potential for long-term ocular sequelae [81]. Similarly, Hashem Abu identified a mean latency of 76.5 days from trauma to the onset of cataract-related symptoms, emphasizing the gradual progression of lens opacification, which in some cases may span several years [80].

The need for screening patients for cataracts following electrical trauma remains an open question. Kaergaard et al. examined 112 individuals with electrical injuries from two Danish registries and found cataract development in 14 cases. Notably, cataracts only occurred when the electrical current came into direct contact with the skull, suggesting that screening may be most relevant for this subset of patients [11]. In this study, among the 55 patients who developed cataracts, 20 had documented entry sites, of whom 14 (70%) had cranial entry and 6 (30%) had non-cranial entry. While this emphasizes the higher prevalence of cataract formation among those with cranial exposure, it also underscores the importance of screening all patients with electrical injuries, regardless of entry site, to detect potential cataract development.

Beyond cataract formation itself, secondary complications such as uveitis may arise due to lens capsule rupture, subsequent protein leakage, or antigen release [82]. However, some studies have reported uveitis occurring in the absence of cataract formation, highlighting the multifactorial nature of intraocular inflammation in electrical injuries [65].

Other complications, such as optic neuropathy, typically manifest over a more extended latency period compared to cataracts, with a mean onset of 156 days post-trauma [80]. Unlike cataracts, where visual loss is potentially reversible, optic neuropathy often leads to permanent vision impairment. In our review, five patients developed optic nerve atrophy, with one reaching no light perception at final follow-up, two with counting fingers vision, and one with hand motion vision, illustrating the poor visual prognosis associated with this condition.

Management of traumatic optic neuropathy (TON) remains controversial, particularly in the context of electrical injuries. High-dose corticosteroids and surgical decompression are the two most frequently considered interventions [83, 84]. However, these studies do not specifically focus on electrical trauma but rather encompass cases of both direct and indirect TON. This lack of targeted research leaves treatment approaches for electrically induced TON unclear, warranting further investigation to establish evidence-based management strategies for this severe complication.

Comparison of ocular manifestations between low-voltage and high-voltage burns is scarce. Bae et al. reported no significant correlation between voltage and the presence of ocular complications (Odd ratio (OR) = 1.536; 95% confidence interval (CI): 0.188–12.550, p = 0.689) or visual disturbances (OR = 2.006; 95% CI: 0.227–17.750, p = 0.532) [4]. Similarly, Han and Park found no significant association between ocular complications and high-voltage electrical injuries or total burn surface area [85]. Furthermore, Ferreriro concluded that voltage does not influence wound severity or the incidence of ophthalmological sequelae (such as cataracts) or non-ophthalmological complications, including limb amputation and neurological disorders [86]. These findings suggest that ocular complications may be independent of current intensity.

In contrast, our study identified variations in the frequency of specific ocular manifestations based on visual impairment severity and voltage exposure. Cataract occurrence increased from 56.00% in patients without visual impairment to 73.68% in those with legal blindness and from 37.50% in low-voltage exposure to 82.35% in high-voltage exposure. Similarly, macular cysts were more prevalent in patients with legal blindness (26.32%) compared to those without visual impairment (12.00%) and only occurred in high-voltage exposures (11.76%). Although these findings suggest that voltage severity may influence the frequency of certain ocular manifestations, they should be interpreted with caution. A potential selection bias exists, as most cases with reported voltage involved high-voltage exposure (17/25, 68.0%). Additionally, information bias may have played a role, as voltage data were not consistently reported—a limitation seen in previous studies due to the rarity of this type of trauma and the inherent difficulty in accurately capturing exact voltage severity [4, 80]. Based on our findings, we suggest that while all patients require thorough ophthalmological evaluation, those exposed to high-voltage injuries or presenting with significant visual impairment should undergo more intensive assessment and follow-up.

Phacoemulsification and vitrectomy were the most frequently performed surgical interventions in cases of electrical and lightning trauma. While specific data on the effectiveness of these procedures in such trauma is lacking, their efficacy in other types of ocular trauma is well-documented. Marcus et al. reported visual improvement in 90% of patients with blunt trauma or penetrating injury after cataract extraction [87]. Similarly, Manjula et al. found that 86% of patients achieved good visual outcomes (final visual acuity between 6/6 and 6/18) following cataract surgery with intraocular lens implantation after trauma-related cataracts [88]. For vitrectomy, Bober et al. demonstrated an improvement in visual acuity in 66% of patients undergoing pars plana vitrectomy for severe ocular trauma (defined as assault and contusion injuries that cause severe visual impairment with a substantial impact on quality of life) [89]. Although these results are not directly comparable to electrical trauma cases, they suggest that these surgeries can significantly improve visual acuity in trauma patients. In our study, 44.64% (25/56) of patients with documented final visual acuity showed no visual impairment; 52.00% (13/25) underwent phacoemulsification, and 8.0% (2/25) underwent vitrectomy.

Final visual acuity in patients exposed to electrical trauma varies significantly, influenced by factors such as trauma severity, timing of medical intervention, and treatment received [2]. Although data specific to electrical trauma is limited, Ozer et al. reported that 69.9% of patients with blunt eye trauma had no visual impairment (defined as 20/50 or better, Snellen chart), while 19.7% experienced some degree of visual impairment (the final visual acuity between 20/200 and 20/50 in the Snellen chart) [90]. In contrast, the present study found that 55.35% (31/56) of patients exposed to electrical trauma and reporting visual outcomes had visual impairment (defined as final BCVA ≥ 20/50 Snellen chart, ≥ 0.4 LogMAR). Among these, 61.29% (19/31) were classified as legally blind (defined as final BCVA > 20/200 Snellen chart, > 1.0 LogMAR) The differences in outcomes may be partly attributed to variations in diagnostic thresholds. Nevertheless, it is crucial to recognize the potentially worse prognosis for patients with electrical and lightning trauma, underscoring the importance of comprehensive examination and closer follow-up based on clinical findings.

This study has some limitations. A primary constraint arises from the fact that most of the included studies are case reports and case series, which inherently carry risks of selection and reporting bias. This limits the ability to calculate prevalence rates using proportion meta-analysis or to establish robust statistical relationships between visual outcomes, voltage severity, and ocular manifestations. Another limitation is that the review may overrepresent severe or unusual cases that are more likely to be published, potentially affecting the generalizability of our findings to broader patient populations. Additionally, while the inclusion of non-English studies increases the diversity of data sources, the reliance on Google Translate for language translation may pose a risk of misinterpretation of complex medical terminology. Finally, crucial information such as entry site of the burn, voltage, or visual acuity is often not recorded, reducing our analysis to 56 cases for final visual acuity and 25 for voltage severity. This issue has also been highlighted by Bae et al. in their cross-sectional study, underscoring the need for better data recording practices [4]. We encourage registries focused on ocular trauma to address this inconsistency in future studies. Despite these limitations, the study provides a comprehensive summary of ophthalmological findings based on more evidence than previous narrative reviews [56, 69, 82]. Therefore, it offers valuable guidance for clinicians in identifying the most common manifestations, visual outcomes, and treatment in patients with electrical trauma.

In conclusion, this systematic review summarized ophthalmological manifestations, visual outcomes, and treatment interventions in electrical and lightning trauma patients. Cataracts, superficial punctate keratitis, and anterior uveitis emerge as the most common anterior segment findings, and macular cysts, macular holes, and RPE disruption are the most common posterior findings. Ocular complications like cataracts have a higher frequency in patients with high-voltage trauma. Surgical interventions like phacoemulsification and vitrectomy are frequently performed, but the specific efficacy of these treatments in the context of electrical trauma remains underexplored. These findings emphasize the importance of thorough ophthalmological evaluation and follow-up in patients exposed to electrical trauma to mitigate long-term visual deficits.

## Supplementary Information

Below is the link to the electronic supplementary material.

Supplementary file1 (DOC 76.5 KB)

Supplementary file2 (DOCX 60.7 KB)

Supplementary file3 (DOCX 630 KB)

## Data Availability

This study is a secondary source analysis that relies on compiling previously published data. Information. Results can be obtained by conducting the search strategy in the databases specified.
