# Supplementary material for: Ophthalmological manifestations, visual outcomes, and treatment of electrical and lightning trauma: A Systematic Review
Source: Graefes Arch Clin Exp Ophthalmol. 2025 Jun 27;263(10):2955–73. doi: 10.1007/s00417-025-06844-3 (PMC12583427; doi:10.1007/s00417-025-06844-3)
Supplement: Supplementary file 2 — Supplementary file2 (DOCX 60.7 KB) [file 417_2025_6844_MOESM2_ESM.docx]

**Supplementary Material 2: Description of cases with electrical trauma and ophthalmological examinations of patients reported until August 2024**

| **Author, year** | **Baseline BCVA** | **Final BCVA** | **Ophthalmological manifestations** | **Medical treatment** | **Surgical Treatment** |
| --- | --- | --- | --- | --- | --- |
| Cherrington et al, 1999 (18) | OD: 1,0 OS: 1,0 | ND | OU: Chemotic conjunctivae, stromal swelling | OU: Cyclopentolate 1% eye drops and topical steroid drops | Not performed |
| Cazabon et al, 2000 (19) | OD: 0,66 OS:1,2 | OD: 0,33 OS: 1,2 | OD: Posterior subcapsular cataract, punctate epithelial erosions | Not performed | OD: Phacoemulsification and IOL implant |
| Sommer et al, 2004 (20) | OD:0,5  OS:0,5 | OD: 1,0 OS: 1,0 | OU: Conjunctivitis, chemosis, corneal edema, increase in IOP | OU: Corticosteroids and tension-lowering drugs | Not performed |
| Lin et al, 2002 (21) | OD:0,8  OS:0,4 | OD: 1 OS: 0,2 | OS: Chemosis, punctate keratitis, corneal edema, anterior uveitis anterior subcapsular lens opacity and macular pigment disruption. | Not performed | OS: Phacoemulsification and IOL implant |
| Espaillat et al, 1998 (23) | OD: 0,2 OS: 0,1 | OD: 0,1 OD: LP | OU: Corneal abrasion, Subcapsular cataract with anterior and posterior component and macular holes, posterior vitreous detachments  OD: Rhegmatogenous retinal detachment | OU: Topical antibiotic ointment | OS: Pars plana vitrectomy, endolaser treatment, fluid-gas exchange, and placement of a scleral buckle |
| Manrique-Cerrillo et al, 2004 (24) | OD: 0,05  OS:0,05 | OD: 0,04  OS:0,5 | Macular cyst | ND | ND |
| Rivas-Aguiño et al, 2006 (25) | OD: 0,13 OS: 0,05 | OD: 0,6 OS: 0,1 | OU: Macular cyst OD: 2+ cells and flare in the anterior chamber OS: Cortical cataract LE | ND | ND |
| Whelan et al, 1988 (26) | ND | ND | OU: Anterior uveitis and cataract unspecified |  | OU: Phacoemulsification and IOL implant |
| Kubilius et al, 2012 (27) | OD: 1 OS: 1 | OD: 0.05 OD: 0.05 | OU: Retinal thickening | ND | ND |
|  | OD: 0,4 OS:1,3 | ND | OS: Cataract | ND | ND |
| Campo et al, 1984 (27) | OD: 0,4 OS: 1,3 | OD: 0,5 OS: 0,66 | OD: Macular hole with macular cystic  OS: Anterior and posterior subcapsular cataract | ND | ND |
| Hunt et al, 2000 (28) | OU: 0.1 | OD:0,3 OS:0,66 | OU: Macular pigment disruption  OD: Anterior uveitis OS: Posterior subcapsular cataract | ND | ND |
|  | OD: 0,2 OS: 0,1 | OU: 1 OD: 0,2 OS: LP | OU: Posterior Vitreous Detachment (PVD), posterior subcapsular cataract and macular holes | Not performed | OS: vitrectomy, endolaser |
|  | OD: 0,4 OS: 1,3 | ND | OU: Posterior subcapsular cataract and macular hole | Not performed | OU: Phacoemulsification and IOL implant |
| Givner et al, 1956  (29) | OD: NLP | ND | OD: Corneal detachment, exposed the iris | Not performed | OD: Evisceration |
| Wainwright et al, 1994 (30) | OD: 0,2  LE: 0,1 | ND | OD: Vitreous bodies  OS: Macular hole secondary to cystic retinal degeneration. | ND | ND |
| Sizman et al, 2020 (31) | OU: 0.05 | ND | OD: Anterior subcapsular cataract OS: Macular Hole | Not performed | OS: Vitrectomy, ILM peeling and perfluorocarbon tamponade |
| Mishulin et al, 2020 (32) | OD: 0.27 OS: CF | ND | OU: Retinoschisis and optic nerve atrophy  OD: Posterior vitreous detachment RE  OS: retinal atrophy | 250 mg acetazolamide per day over 3 months and prednisolone 100 mg per day for 1 week. | Not performed |
| Almari et al, 2020 (33) | OD: 0.2 OS: 0.1 | ND | OU: Corneal edema, superficial punctate keratitis, conjunctival chemosis, unspecified anterior uveitis, increased intraocular pressure, anterior subcapsular cataract and macular hole | OU: Prednisolone acetate 1%, brimonidine and dorzolamide and atropine sulfate 1% over 3 months. | Not performed |
| Korkmaz et al, 2018 (34) | OD: 0.3 LE: Hand motion | ND | OD: Cortico-nuclear cataract and full-thickness macular hole  OS: Mature cataract and foveal atrophy | ND | OS: cataract surgery |
| Pradhan et al, 2020 (35) | ND | ND | OU: Conjunctival chemosis, cataract and hazy cornea | OU: Topical antibiotics, cycloplegics and steroids. | Phacoemulsification + IOL implant |
|  | OU: 0,.3 | OD: 0.5  OS 0.62 | OU: Parafoveolar cysts | OU: Topical corticosteroids | Not performed |
|  | OU: 0.16 | OD: 0.25  OS: 0.5 | OU: Parafoveolar cysts | OU: Topical corticosteroids and cycloplegics | Not performed |
|  | OD: 0.66 OS: 0.1 | OS: 0.5 | OS: Parafoveolar cysts | Topical and systemic corticosteroid (prednisolone 1mg/kg) for 4 weeks | Not performed |
|  | OU: Hand motion | ND | OU: Conjunctival congestion, uveitis anterior, parafoveolar cysts with foveoschisis-like lesions. | Topical and systemic corticosteroid (prednisolone 1mg/kg) + cycloplegic | Not performed |
|  | OD: 0,16, LE: 1.0 | OD: 0.33 | OD: Parafoveolar cysts | OD: Topical and cycloplegic corticosteroid | Not performed |
|  | OD: Hand motion OS: 0.66 | ND | OD: Corneal edema. foveal cyst, optic neuropathy. posterior subcapsular cataract | OD: Topical corticosteroids and cycloplegics | Not performed |
| Yadav et al, 2020 (36) | OU: PL | ND | OU: Total cataract | Not performed | OU: Phacoemulsification + IOL implant |
| Harris et al, 2019 (37) | OD: 0.25 OS: NPL | ND | OU: Cataract with severe bilateral choroidal atrophy of the posterior pole | Not performed | OU: Cataract extraction |
| Kumawat et al, 2017 (38) | OU: counting fingers | OU: 0.66 | OD: Nucleus was dislocated posteriorly into the vitreous cavity with a nuclear cataract  OS: Anterior subcapsular cataract along with nuclear sclerosis | Not performed | OD: Pars plana vitrectomy, removal of nucleus, insertion of IOL implant in sulcus |
| Liu et al, 2016 (39) | OD: 0.2 LE: 1.0 | OD: 0.5 | OD: Posterior subcapsular cataract + mild cystic macular edema RE | ND | ND |
| Izzy et al, 2014 (40) | OS: 0.5 | ND | OD: Optic nerve atrophy | Methylprednisolone 1 g IV for 3 days | Not performed |
| Korn et al, 2014 (22) | OU: Hand motion | ND | OU: Anterior subcapsular opacities of the lens, optic nerve atrophy and generalized macular pigment disruption | Not performed | OU: Phacoemulsification + IOL implant |
| Baranwal et al, 2012 (41) | OD: 0.33 OS: 0.25 | OD: LP OS: 1.0 | OU: Anterior subcapsular cataract | Not performed | OS: Phacoemulsification + IOL implant under peribulbar anesthesia |
| Bayar et al, 2013 (42) | OD: 0.05 OS: 1.0 | ND | OD: Optic atrophy | Methylprednisolone 1000 mg/day for 3 days | Not performed |
| [Toprak et al, 2014 (43)](https://www.zotero.org/google-docs/?r0QMvG) | OD: 0.4 OS: 0.5 | ND | OU: Cortical and posterior lens opacities + macular pigment disruption | ND | Not performed |
| Tandon et al, 2014 (43) | OD: PL  OS: CD | OU: CF | OU: Retinal thickening  OS: Corneal congestion, superficial punctate keratitis and cataract | Prednisolone 30 mg/day and acetazolamide 750 mg/day for 1 month. | OU: Phacoemulsification |
| Armstrong et al, 2010 (44) | OD: 0.4  OS:0,66 | ND | OS: Atrophic fovea | ND | ND |
| Rajagopal et al, 2010 (45) | OD; 0,66 OS: 0,33 | ND | OS: Macular hole, PVD and RD | Not performed | OS: Pars plana vitrectomy |
| Rao et al, 2009 (46) | OD: 0,5 OS:1 | ND | OU: Posterior subcapsular lens opacities with full thickness macular hole  OD: Anterior uveitis | OU: Topical and cycloplegic steroids | Not performed |
| Khan et al, 2009 (47) | OD: 0,36  OS: 0,77 | ND | OD: Cataract and optic nerve atrophy | ND | ND |
| Grewal et al, 2007 (48) | OD:0,25  OS: 0,29 | ND | OS: Anterior subcapsular lenticular opacities and increased IOP | Not performed | OS: phacoemulsification surgery and IOL implant |
| Liyanage et al, 2006 (49) | OD: 0,5  OS: MM | ND | OU: Cataract, macular cyst and anterior uveitis | ND | ND |
| Mutlu et al, 2004 (50) | OD: MM  OS: CD | ND | OU: Non granulomatous anterior uveitis  OS: Cataract subcapsular posterior | Not performed | OU: phacoemulsification surgery and IOL implant |
| Dinakaran et al, 1998 (51) | OU: PL | ND | OD: Posterior subcapsular cataract | Not performed | OD: Yag laser capsulotomy, extracapsular cataract extraction without IOL implant |
| Ranjan et al, 2017 (52) | OS: 0,17  OS: 0,25 | ND | OU Electric shock maculopathy with spontaneous recovery | ND | Not performed |
| Dolphin et al, 1992 (53) | OD: 0,2  OS: 0,5 | ND | OU: Corneas had a broad band of microcystic edema; juxtafoveal oval white lesions (scotomas) with spontaneous resolution | ND | Not performed |
| Khazaei et all, 2022 (54) | OU: 0,05 | ND | OD: Macular edema secondary to CRVO | ND | Not performed |
| Dhillon et all, 2015 (55) | OD:NPL  OS: MM | ND | OD: Macular edema and macular hole  Topical NSAID (allergy) | Not performed | Referred to a tertiary center for macular hole surgery |
| Moon et al, 2005 (56) | OU: 0,05 | ND | OU: Cataract posterior subcapsular and lightning maculopathy | Not performed | OD: Cataract surgery and IOL implant |
| Choe et al, 2019 (57) | OD: 0,05  OS: 0,66 | ND | OS: Bilateral cataract and retinal atrophy | Not performed | Cataract extraction and IOL implant. |
| Faustino et al, 2014 (58) | OD: CF  OS:1,0 | ND | OU: Periorbital edema, conjunctival chemosis, mild corneal edema with diffuse punctate keratitis, retinal cyst in inner nuclear layer, retinal detachment (3) with macular holes and cataract | 1.Pomade containing 10,000 IU of retinol acetate and artificial tears | OU: pars plana vitrectomy and just one with silicone oil  Phacoemulsification with IOL implant |
| Hashemi et al, 2008 (59) | OD: Hand motion | ND | OU White cataracts | Not performed | OU: phacoemulsification and IOL implant |
| Sony et al, 2005 (60) | OD:20/125 OS: 20/80 | ND | OU: Macular hole  OD: Macular hole and a few punctate lenticular opacities | ND | ND |
| Ouyang et al, 2014 (61) | OD: CD  OS: 0,2 | ND | OU: Anterior subcapsular opacities in the lens, vitreous detachment, retinal hemorrhages | Not performed | OD: Pars plana vitrectomy + internal limiting membrane peeling + C3F8 gas tamponade with face-down positioning and, finally, an intraocular injection of 0.05ml (2mg) triamcinolone acetonide |
| Lakosha et al, 2009 (62) | OU: 0.5 | ND | OU: Anterior uveitis and cataract | OU: Steroid and cycloplegic drops | Not performed |
| Gupta et al, 2009 (63) | OU: 0,05 | ND | OU: Anterior and posterior subcapsular cataract and posterior vitreous detachment | Not performed | OU: phacoemulsification surgery and IOL implant |
| Al Rabiah et al, 1987 (64) | OD:NPL  OS: MM | ND | OU: Cataract  OD: Upper and lower eyelids burned and charred and symblepharon, corneal opacification, synechiae and coagulative necrosis of the cornea, sclera and anterior uveal tract  OS: Ectropion of the left upper eyelid, atrophic optic disc and macular pigmentary degenerative | ND | ND |
| Biro et al, 1994 (65) | OU: 0,05 | ND | OU: Optic neuritis and cataract, several, subcapsular, patchy lens opacities were revealed. The nucleus was opaque as well as the posterior cortex. | Not performed | OU: Extracapsular cataract extraction with primary PC-IOL implant |
| Pineda-Vanegas et al, 2024 (70) | OD: 0,05  OS: 0,66 | ND | OD: Purtscher‐like retinopathy OS: anterior uveitis and hyphema | OS: Topical steroids and mydriatics | Not performed |
| Reddy et al, 1999 (67) | OD: CD  OS :1,0 | ND | OU: Anterior subcapsular cataract OD: Anterior subcapsular ring-like opacity in the periphery with irregular margins  OS: Diffuse opacity with opacification of the nucleus and cortex | ND | OU: manual extracapsular cataract extraction with 6 weeks of differences. |
| Duman et al, 2015 (70) | OD: MM | ND | OD: Non-granulomatous anterior uveitis, nuclear cataract and retinal detachment. | Topical dexamethasone and %1 cyclopentolate HCL ophthalmic solution four times daily. | OD: Vitreoretinal and cataract surgery. |
| Peñaranda C, 2016 (71) | OU: 0.5 | ND | OU: Anterior subcapsular lenticular opacities and macular lesions | Vitamin C Calcium dobesilate was administered-. | Not performed |
| Zhang et al, 2023 (72) | OD: 0.25  OS: 1.0 | OD: NPL | OS: Necrosis of iris, ciliary epithelium, retina and choroid, and central corneal epithelial defect with corneal edema and anterior uveitis | Not performed | OS: Evisceration reconstruction of eyelids and eye socket |
| Bae et al, 2013 (4) | ND | ND | Electric cataract 7  Eyelid erythema, swelling 5  Conjunctivitis 4  Macular edema 4  Foveal cyst 1  RD 1  Pseudohole 1  Uveitis 2  Corneal problems: PEE 16  ED 2  Filament keratitis 2  Corneal FB 2  Corneal stromal opacity 1 Exposure keratitis 2  Corneal scarring 1 | ND | ND |
| Pineda-Vanegas et al, 2024 (73) | OD: 6/120 OS: 1.3 | OD: 0.17 OS: 0 | OD: Conjunctival hyperemia, moderate corneal oedema, mild Descemet’s membrane folds, 2+ cells in the anterior chamber, irregular mid-dilated and non-reactive pupil | Tropicamide 1%, prednisolone 1%, dexamethasone 8 mg | Not performed |
| Venkateswaran, et al 2018 | ND | ND | OD: rosette-shaped cataract and posterior vitreous detachment  OS: ND | ND | ND |
| Rishi et al, 2016 | OD: 6/7.5  OS: 6/6 | ND | OD: Normal  OS: hole at macula with intraretinal cyst  and a group of pigment clumps arranged in a wedge‐shaped pattern in the temporal periphery of retina. | ND | ND |
| Walkow et al, 2011 | OD: 0.4  OS: 1 | OD: 0.4  OS: 1 | OD: macular hole, optic atrophy, electric cataract and keratoconjunctivitis sicca and cataract.  OS: Cataract | ND | OU: Cataract extraction and IOL implant and vitrectomy |
| Boozalis et al, 1991 | OD: HM  OS: 0.48 | ND | OD: Keratic precipitates, 1+ cell and flare, anterior subcapsular opacities.  OS: anterior subcapsular opacities | OD: Hyoscine and Pred forte | OU: Cataract extraction |
|  | OD: 0.20  OS: 0.1 | ND | ND | ND | ND |
|  | OD: 1  OS: 0.1 | ND | OD: cataract  OS: cataract | ND | OU: Cataract extraction |
|  | ND |  | OD: conjunctival chemosis and trace corneal edema. Cortical cataract.  OS: conjunctival chemosis. Cortical cataract. | ND | ND |
|  | OD: LP  OS: 0.7 | OD: LP  OS: 1.3 | OD: trace corneal edema, pale atrophic disc, cortical cataract  OS: Macular hole, cortical cataract | ND | ND |
| Zhang et al, 2024 | OD: 0  OS: 0.25 | ND | OD: normal  OS: large central corneal epithelial defect with  corneal edema and 3+cell/flare in the anterior chamber. perforating scleral wound with extraocular extension  of vitreous, retina, choroid, and ciliary epithelium. Acute keratitis, hypopyon, necrosis of the superior iris, ciliary body, retina, retinal pigment epithelium,  choroid, periocular connective tissue and extraocular muscle, and chronic non-granulomatous uveitis were present. | ND | Evisceration |
| Yi et al, 2024 | OD: HM  OS: HM | ND | OD: Corneal edema, anterior chamber flare, cataract  OS: Corneal edema, anterior chamber flare, cataract | topical antibiotic eye drops, tobramycin dexamethasone eye drops | ND |
| Valera-cornejo et al, 2020 | OD: 1.3  OS: CF | OD: 1.3  OS: CF | OD: cataract, chorioretinal atrophy, RPE disrruption  OS: cataract, chorioretinal atroph, RPE disrruption | ND | OU: phacoemulsification with intraocular lens implantation |
| Khadka et al, 2021 | OD: 0.5  OS: 1 | OD: 0.2  OS: 1 | OD: Macular hole, macular cysts. cortical and posterior subcapsular cataract  OS: 1+ anterior chamber cells. Macular hole, macular cyst. cortical and posterior subcapsular cataract | topical nonsteroidal anti-inflammatory drugs | ND |
|  | OD: 0.6  OS: 0.5 | OD: 0.6  OS: 0.3 | OD: 2+ AC cells. Intraretinal cystoid abnormalitie    OS: 1+ AC cells. Intraretinal cystoid abnormalitie | topical steroid and cycloplegic agent | ND |
| Datta et al, 2002 | OS | OS | ND | ND | ND |
| Bienfang et al, 1980 | OD: | OD: | ND | ND | ND |
| Hashemi et al, 2008 | OS | OS | ND | ND | ND |

OD: Right eye, OS: Left eye, OU: Both eyes, ND: No data, LP: Light perception, NLP: No light perception, CF: Counting fingers, IOL: Intraocular lens, RD: Retinal detachment, PEE: punctate epithelial erosion, ED: Epithelial defect, FB: Foreign body, ED: Epithelial defect CRV: Central retinal vein occlusion.
