# Supplementary material for: Ophthalmological manifestations, visual outcomes, and treatment of electrical and lightning trauma: A Systematic Review
Source: Graefes Arch Clin Exp Ophthalmol. 2025 Jun 27;263(10):2955–73. doi: 10.1007/s00417-025-06844-3 (PMC12583427; doi:10.1007/s00417-025-06844-3)
Supplement: Supplementary file 3 — Supplementary file3 (DOCX 630 KB) [file 417_2025_6844_MOESM3_ESM.docx]

Supplementary Material 3

**Figure 1: Risk of bias of the risk of bias in the case report and series included using the Hassan Murad tool**


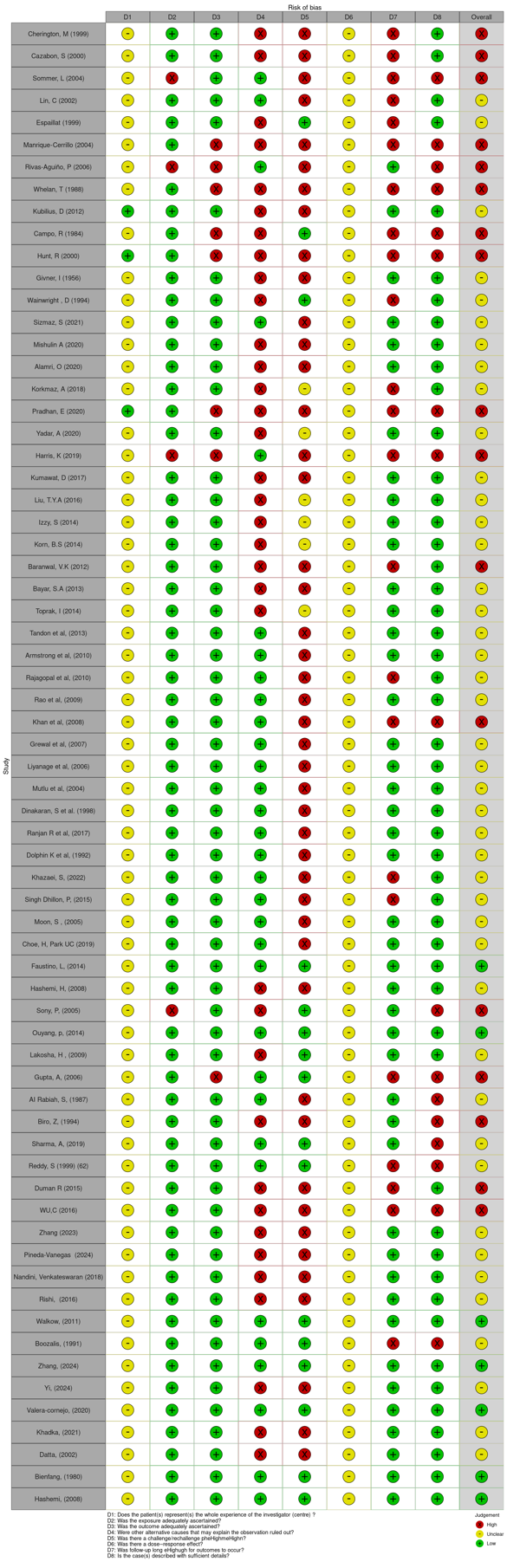


**Figure 2: Risk of bias of the risk of bias in the cross-sectional study included using the Hoy et al. tool.**

**
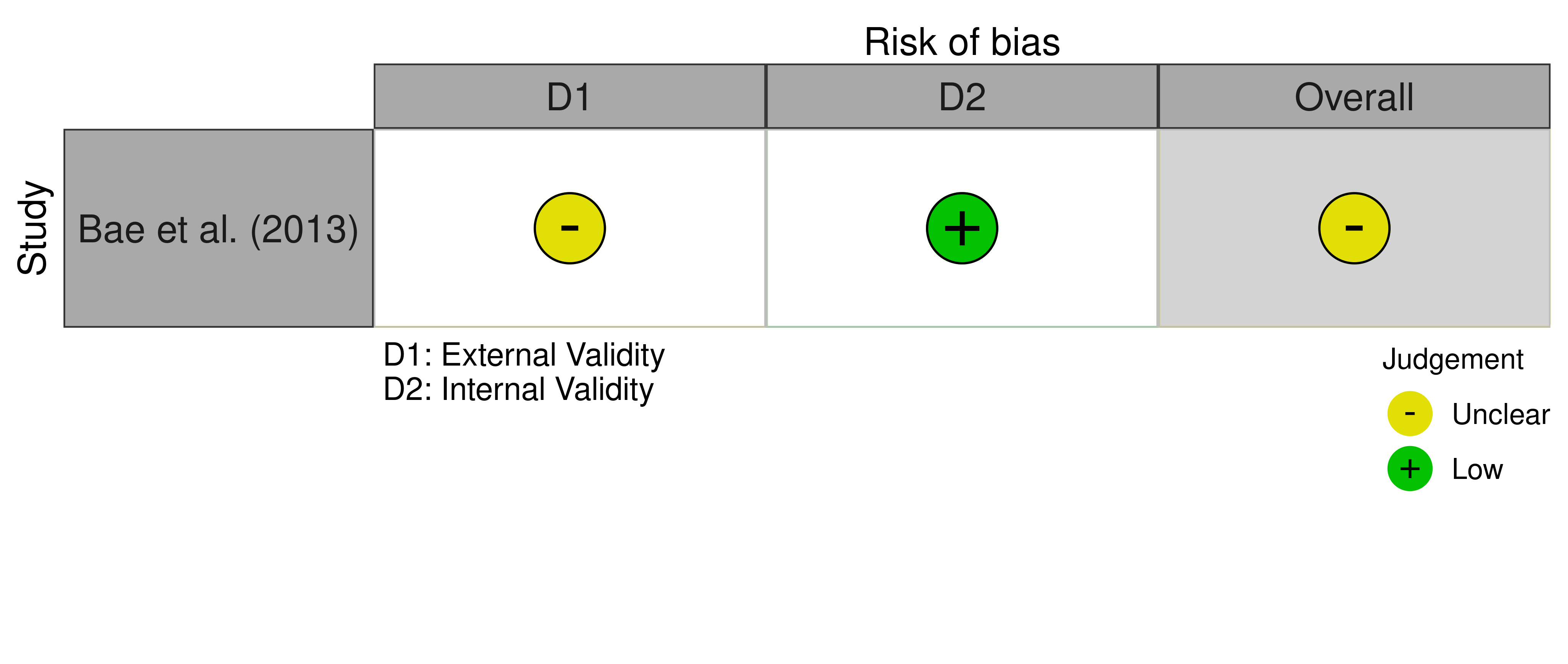
**
